# INFLUENCE OF SATISFACTION WITH INTERVENTIONS ON THE OCCURRENCE OF FALLS IN OLDER PEOPLE: RANDOMIZED CLINICAL TRIAL

**DOI:** 10.1093/geroni/igae098.3367

**Published:** 2024-12-31

**Authors:** Juliana Ansai, Leticia Teodoro Maciel, Mariana Ignácio Sossai, Mariana Luiz de Melo, Graziele Norberto Pereira, Mel Silva de sá, Ana Beatriz Simões Pereira, Karina Gramani-Say

**Affiliations:** Federal University of São Carlos, São Carlos, Sao Paulo, Brazil; Federal University of São Carlos, São Carlos, Sao Paulo, Brazil; Federal University of São Carlos, São Carlos, Sao Paulo, Brazil; Federal University of São Carlos, São Carlos, Sao Paulo, Brazil; Federal University of São Carlos, São Carlos, Sao Paulo, Brazil; Federal University of São Carlos, São Carlos, Sao Paulo, Brazil; Federal University of São Carlos, São Carlos, Sao Paulo, Brazil; Federal University of São Carlos, São Carlos, Sao Paulo, Brazil

## Abstract

Falls are recurrent among older people, and there is a need of more personalized interventions to be adopted to prevent falls. The purpose of this study was to analyze the influence of satisfaction with the interventions on occurrence of falls in older people with a history of falls. This is a randomized clinical trial, with older people with at least 2 falls in the past year. Participants were allocated into Control Group (CG) (with monthly monitoring about falls and general health) and Intervention Group (IG). The IG was submitted to a case management intervention over 16 weeks, including a multidimensional assessment, intervention proposal and individualized care plan. The assessment consisted of falls data (falls calendar and monthly phone calls) over 16 weeks and satisfaction with interventions (Short Assessment of Patient Satisfaction SAPS 0-28, and overall satisfaction with care 0-10) after 16 weeks. The final sample consisted of 56 older people. The median SAPS score in the GC was 22.00 and in the IG 25.00 (p=0.046). The median overall satisfaction score was 10.00 in both groups (p=0.782). After 16 weeks, 34.5% of the IG and 48.1% of the CG fell (p>0.05), and 6.9% of the IG and 14.8% presented harmful falls (p>0.05). There was no influence of satisfaction with intervention on falls data over the short follow-up. Future studies that identify other factors related to improvements in falls data among brazilian faller older people are needed.

